# Public stigma towards prolonged grief disorder: Does diagnostic labeling matter?

**DOI:** 10.1371/journal.pone.0237021

**Published:** 2020-09-11

**Authors:** Judith Gonschor, Maarten C. Eisma, Antonia Barke, Bettina K. Doering

**Affiliations:** 1 Clinical Psychology and Psychotherapy, Department of Psychology, Philipps-University Marburg, Marburg, Germany; 2 Department of Clinical Psychology and Experimental Psychopathology, University of Groningen, Groningen, the Netherlands; 3 Clinical and Biological Psychology, Department of Psychology, Catholic University Eichstaett-Ingolstadt, Eichstaett, Germany; Universitat d'Alacante, SPAIN

## Abstract

The recent introduction of prolonged grief disorder (PGD) as a diagnostic category may cause negative social reactions (i.e. public stigma). Vignette experiments demonstrate that persons with both PGD symptoms and a PGD diagnosis elicit more public stigma than persons who experience integrated grief. However, the strength of the influence of the diagnosis itself remains unclear: We aimed to clarify if the diagnostic label PGD produces additional public stigma beyond PGD symptoms. We further compared whether public stigma varies between the label PGD and the label major depressive episode (MDE) (when PGD symptoms are present) and if gender of the bereaved person influences public stigma or moderates the aforementioned effects. Eight-hundred fifty-two participants (77% female; *M*_age_ = 32.6 years, *SD* = 13.3) were randomly assigned to read online one of eight vignettes describing either a bereaved male or female, with PGD symptoms and PGD diagnosis; PGD symptoms and MDE diagnosis; PGD symptoms and no diagnosis, or no PGD symptoms and no diagnosis (i.e., integrated grief). Following the vignettes, participants indicated which negative characteristics they ascribed to the person, their emotional reactions, and preferred social distance from the person. People with PGD symptoms and PGD (or MDE) diagnosis were attributed more negative characteristics, and elicited more negative emotions and a stronger desire for social distance than people with integrated grief. However, public stigma did not differ for people with both PGD symptoms and diagnosis compared to people only experiencing PGD symptoms. Gender of the bereaved only had an influence on desired social distance, which was larger towards men. Helping severely distressed bereaved people (regardless of diagnostic status) cope with negative social reactions may help them adapt to bereavement. Results demonstrate that the experience of severe grief reactions, yet not a diagnostic label per se, causes public stigma.

## Introduction

The loss of a loved one is a near-universal experience. Whilst often accompanied by emotional upheaval, the majority of bereaved persons cope with their loss with the help of internal resources; e.g. emotion regulation strategies and external support [1]. Thereby, acute grief is assumed to eventually evolve into ‘integrated grief’, the permanent response after adaptation to the loss, in which satisfaction in ongoing life is renewed [2, p2]. A minority develops severe, disabling and protracted grief reactions [3]. Concepts such as ‘complicated grief’ [4] or ‘prolonged grief’ [5] describe such grief patterns with varying diagnostic criteria. Previous research consistently demonstrates that such non-normative grief patterns are associated with severe mental health impairments (e.g., higher depression rates and suicidal tendencies) and reduced quality of life (e.g., [6]).

Recently, the World Health Organization (WHO) has acknowledged prolonged grief disorder (PGD) in the revised classification system ‘International Statistical Classification of Diseases and Related Health Problems’ (ICD-11) as a distinct mental health condition among disorders specifically associated with stress [7]. Core symptoms of PGD are (1) persistent and pervasive longing for the deceased and (2) persistent and pervasive preoccupation with the deceased. At least one core symptom and one of ten accessory symptoms indicative of emotional distress, including difficulty accepting the death, inability to experience positive mood, and social withdrawal have to persist for at least 6 months to diagnose PGD. The grief reaction must exceed relevant social and cultural norms and cause clinically significant impairment. Estimates of prevalence rates of prolonged grief reactions vary depending on cause of death. Recent meta-analyses estimated its prevalence to be approximately 10% among persons who experience non-violent bereavement [3], and 49% for people experiencing violent loss [8]. Kersting et al. [9] reported a conditional prevalence of 7% after the death of a significant other in a German representative population-based sample.

Classifying mental health phenomena such as prolonged grief reactions as disorders has important consequences on both the individual and the societal level. On the one hand, recognition of a phenomenon as disorder can facilitate provision and access to effective treatments [10]. On the other hand, it carries the risk of stigmatization [11].

In the context of mental health, researchers distinguish two interacting levels of stigma: public stigma and self-stigma. Public stigma has been defined as ‘the phenomenon of large social groups endorsing stereotypes about and acting against a stigmatized group’ [12 p179]; e.g., against people suffering from mental illness. Self-stigma occurs when affected individuals internalize public stigma and come to believe that they are less valuable because of their disorder in the same way as they are described by others [12]. Socio-cognitive models of public stigma propose that stigma occurs in stages: It begins with the acquaintance with stereotypes (e.g., ‘People with mental illness are dangerous’). The agreement with a certain stereotype in turn leads to prejudice, which is accompanied by an emotional reaction towards a certain group (e.g., ‘People with mental illness scare me!’). Such negative cognitive and affective evaluation can result in discriminatory behavior such as actions of the stigmatizing group that reduce opportunities for work and housing for the stigmatized group [12, 13] or result in withholding help, social avoidance, and coercive treatment [14].

In addition to possible discrimination, public stigma towards persons with mental illness can have a negative impact on the effectiveness of prevention of mental disorders [15], and attitudes towards seeking professional help [16]. Most population-based stigma studies deal with public stigma in schizophrenia and major depression (MDE) [e.g., 17, 18]. Public stigma research is typically conducted using self-report questionnaires, e.g., by asking participants about their attitudes towards people with a certain mental illness. An alternative approach is offered by vignette experiments, which offer both the possibility to standardize the presentation of the mental illness by presenting participants with a description of a person suffering from the mental health condition, and to systematically vary these descriptions in order to assess emotional reactions or attitudes toward the different descriptions.

With regard to PGD, we consider stigma especially relevant. This is because stigma may lead to a decline in social support, which is considered an important factor in coping with bereavement [19]. Regardless of diagnostic status, grief severity itself seems to be an important factor contributing to stigmatizing reactions towards bereaved persons: Johnson et al. [20] demonstrated that individuals with prolonged grief reactions who had not received any diagnosis experienced and expected more negative responses from their social environment (i.e. more perceived stigma). Relatedly, Kahler et al. [21] found that higher grief severity in a vignette (no diagnosis mentioned) was associated with greater reported social discomfort towards the bereaved person described in the vignette (i.e., more public stigma).

Apart from grief severity, diagnostic labeling appears an important factor in stigmatization. One argument against the introduction of PGD as a diagnosis is the fear, voiced by practitioners, researchers, and lay people alike, that the introduction of PGD as diagnostic category may cause stigma and could thereby additionally burden affected people [13, 22, 23]. The stigma studies mentioned above do not answer this important question: What (additional) harm does the diagnostic label PGD do in the presence of severe grief reactions?

Eisma [22] and Eisma et al. [23] have shed more light on this issue by conducting vignette-based experiments among participants from the general public using comprehensive assessments of public stigma. These experiments demonstrated that people with PGD (vs. without) are attributed more negative characteristics, elicit more negative emotions, and a larger preferred social distance. These results seem to suggest that the mere presence of a PGD diagnosis elicits public stigma. However, in these experiments the PGD diagnosis and PGD symptoms were always presented simultaneously. Thus, we cannot be sure whether the observed stronger public stigma for PGD in these experiments is due to the specific diagnosis of PGD, the presence or severity of the described symptoms, the labeling of a person as suffering from a mental health condition, or any combination of these factors. Therefore, the first aim of the present study was to disentangle the effects of a PGD label vs. symptoms on public stigma by systematically varying the experimental factors ‘presence of PGD symptoms’ and ‘diagnosis of a mental health condition’.

The second aim of our study was to investigate how ‘harmful’, i.e. stigmatizing, the label of a PGD diagnosis is, compared to other diagnostic labels. Previous research on public stigma has shown that public stigma depends on the mental health condition under study [17, 24–28]. Persons with depression or anxiety disorders, for example, elicit lower stigmatizing responses than persons with schizophrenia or alcohol/substance abuse [17, 24, 26]. Arbanas, Rožman and Bagari [27] compared public stigma towards depression (MDE) and posttraumatic stress disorder (PTSD), which is—just like PGD—a disorder connected to an external cause. The authors did not use the vignette method and only provided the diagnostic label and no further description. Respondents rated a set of items assessing negative attributes, emotional reactions and social distance. They answered these items twice in randomized order, first with regard to a person diagnosed with depression and secondly with regard to a person diagnosed with PTSD. In that study, lay people reported no difference on stigma-related variables between labels. On the other hand, Feldman and Crandall [28] have shown that preferred social distance was lower toward people with PTSD when compared to people with MDE. In their study, the authors presented vignettes describing the unique symptoms of each condition, a label and a brief definition of the disorder. Since stigma thus potentially varies between diagnoses, we chose to include a comparator when investigating the public stigma of PGD. Our choice to include MDE as the relevant comparator was based on two reasons. On the one hand, we were confident that the label MDE could be applied to a description of PGD symptoms rather naturally without creating confusion. Before the introduction of a grief-specific diagnostic entity (PGD), clinicians often classified bereavement-related psychological pathologies as MDE for different reasons [29, 30] such as the apparent similarities of the respective symptoms. On the other hand, the well-known and extensively investigated public stigma brought about by the label MDE provides us with a relevant reference frame to evaluate the harmfulness of the label PGD. Looking at public stigma elicited by the label PGD, we were especially interested in comparing stigmatizing effects between the labels MDE and PGD whilst keeping the presented symptomatology constant. To the best of our knowledge, no research has yet compared the public stigma of the PGD diagnostic label to other diagnostic labels.

Third, we were interested in the effect of gender of the bereaved on stigmatization of bereaved people with or without severe mental health problems after loss. A meta-analysis of Parcesepe and Cabassa [26] across different mental health conditions reported no influence of gender of the person suffering from a mental illness on public stigma. Evidence from more grief-specific research is inconclusive: One vignette study that did not provide any information on grief severity of the bereaved person, demonstrated that a male person elicited a stronger desire for social distance than a female person when the type of death was a stroke [31]. Similar, Kubitz, Thornton and Robertson [32] found that when the vignette described a sudden death, participants were more willing to interact with a female than a male bereaved person. In this study, however, the effect was only evident for vignettes describing high grief intensity (vs. low grief intensity).

Targeting primarily attitudes towards non-pathological grief, Versalle and McDowell [33] found no differences in sympathy for male vs. female grievers. Logan, Thornton, Kane and Breen [34] also reported no effect of gender on likeability of the bereaved, blame attributions and behavioral intentions. A review by Logan et al. [35] reported mixed results with some studies showing that bereaved women were offered more social support and other studies demonstrating no such effect. Studies of gender effects on stigma in non-normative grief patterns (e.g., PGD) are lacking.

To summarize, our study used a vignette experiment to cross-validate the findings of Eisma [22] and Eisma et al. [23] on public stigma for PGD, and expand on these findings by examining the effects of diagnostic labeling and gender of the bereaved. We had the following hypotheses: (1) A person with PGD symptoms and a mental health diagnosis (PGD or MDE) evokes more public stigma than a person with integrated grief (i.e., no PGD symptoms and no diagnosis). (2) A person with PGD symptoms and a mental health diagnosis (PGD or MDE) elicits more public stigma than a person with only PGD symptoms. We further explored the following questions: (3) When PGD symptoms are present, does public stigma differ between persons with PGD diagnosis and MDE diagnosis? (4) Does the gender of the bereaved influence (or (5) modulate group differences in) public stigma? Drawing on former research in non-pathological grief, we suspected a higher desire for social distance towards bereaved men than women. This effect might only be present in response to vignettes presenting integrated grief.

## Materials and methods

### Ethical statement

The ethics committee at the Department of Psychology, Philipps-University Marburg, approved this study (2018-21k). The study was conducted according to the principles expressed in the Declaration of Helsinki [36]. All participants provided written informed consent.

### Recruitment and procedure

A convenience sample of the general public was recruited. Exclusion criteria were age under 18 years and insufficient knowledge of the German language (assessed via self-report). Recruitment took place using a variety of strategies to ensure that a wide range of people read the study advertisement; Recruitment took place online (Facebook groups, university student and general staff mailing lists), via an article in a local newspaper reporting on bereavement research, and advertisements in public places(e.g., bus stops, city offices).

Respondents were invited to access the study website directly via a web-link or QR-Code. The study was conducted online and programmed in Unipark Questback. Respondents were informed that the aim of the study was to gain knowledge about the public’s attitude, feelings and intended behavior towards bereaved people in general. We made sure to prevent presenting any information on stigma or grief disorders. We further provided information about the study procedure (e.g., voluntariness of participation, data handling). Next, people were asked to provide informed consent.

They provided basic demographic information (gender, age, educational level) and answered questions regarding personal experiences of bereavement. Respondents who had personally experienced a bereavement also filled in the Inventory of Complicated Grief (ICG [37]; German version: [38]). All respondents were subsequently randomly assigned to read one of eight vignettes, describing a bereaved individual (see Table 1). Next, they answered questions about indicators of public stigma, namely the personality characteristics attributed to the person, their emotional reactions towards the person, and their own desire for social distance. Afterwards, they briefly answered questions to check whether they had understood the vignette content, which served as a manipulation check. At the end of the survey, participants could participate in a prize draw for vouchers of a popular online store. Mean time to answer the survey was *M* = 11.8 min. (*SD* = 7.2 min.).

**Table 1:** Content of 8 vignettes varying ‘mental health condition’ and ‘gender of bereaved person described’.

	**Vignettes 1 [*Carl*] and 2 [*Ruth*]**
**PGD symptoms and PGD diagnosis**	50-year old *Carl/Ruth* has lost *his wife/her husband* more than two years ago. *He/She* finds this very difficult and no longer functions well at work and at home. Since the loss, *he/she* yearns strongly for *his/her* lost *wife/husband*. *Carl/Ruth* has trouble accepting the loss and has strong feelings of guilt. *He/She* withdraws socially and undertakes very few activities. *Carl/Ruth* has visited a psychotherapist and discussed *his/her* situation and feelings with him. On the basis of *his/her* behavior he diagnosed *him/her* with a prolonged grief disorder.
	**Vignettes 3 [*Carl*] and 4 [*Ruth*]**
**PGD symptomsand MDE diagnosis**	50-year old *Carl/Ruth* has lost *his wife/her husband* more than two years ago. *He/She* finds this very difficult and no longer functions well at work and at home. Since the loss, *he/she* yearns strongly for *his/her* lost *wife/husband*. *Carl/Ruth* has trouble accepting the loss and has strong feelings of guilt. *He/She* withdraws socially and undertakes very few activities. *Carl/Ruth* has visited a psychotherapist and discussed *his/her* situation and feelings with him. On the basis of *his/her* behavior he diagnosed *him/her* with a depressive episode.
	**Vignettes 5 [*Carl*] and 6 [*Ruth*]**
**PGD symptoms and no diagnosis**	50-year old *Carl/Ruth* has lost *his wife/her husband* more than two years ago. *He/She* finds this very difficult and no longer functions well at work and at home. Since the loss, *he/she* yearns strongly for *his/her* lost *wife/husband*. *Carl/Ruth* has trouble accepting the loss and has strong feelings of guilt. *He/She* withdraws socially and undertakes very few activities. *Carl/Ruth* has visited a psychotherapist and discussed *his/her* situation and feelings with him. On the basis of *his/her* behavior he provided *him/her* with information about grief symptoms.
	**Vignettes 7 [*Carl*] and 8 [*Ruth*]**
**No symptoms and no diagnosis**	Fifty year-old *Carl/Ruth* has lost *his wife/her husband* to a stroke around two years ago. While *he/she* was very sad the first few months after the loss, *he/she* now has learned to live with the loss. *He/she* functions well both at work and at home. *He/She* has accepted the loss of *his wife/her husband* more, occasionally engages in fond reminisces of *her/him* and feels *his/her* life is meaningful. *Carl/Ruth* has begun to engage in some new hobbies and talks about *his wife/her husband* now and then to *his/her* close friends.

### Vignettes

Respondents were randomly assigned to read one of eight vignettes, which varied on the independent variables ‘gender of bereaved person described’ (female = ‘Ruth’ vs. male = ‘Carl’) and ‘mental health condition’ (PGD symptoms and PGD diagnosis *vs*. PGD symptoms and MDE diagnosis *vs*. PGD symptoms and no diagnosis *vs*. no symptoms and no diagnosis). Vignettes were identical on all other accounts (see Table 1). The vignette content was based on the research of Eisma [22] and Eisma et al. [23]. We pretested vignettes with four research experts in the field of bereavement to ensure the vignettes’ content validity. For those vignettes presenting PGD symptoms, vignettes met ICD-11 criteria of PGD [7]. Vignettes explicitly named the following criteria: Intense emotional pain (yearning), trouble accepting the loss, feelings of guilt, difficulty in engaging with social or other activities, time since loss over 6 months (‘around two years ago’) and impairment in functioning. Vignettes describing a person with integrated grief described the same time since loss but explicitly mentioned that there was no persistent, intense emotional pain or functional impairment, but that there was acceptance of the loss and pursuit of social activities.

### Questionnaires

#### Demographic and bereavement-related variables

Respondents provided basic demographics on gender (male, female, other), age (in years) and educational level. For the description of the sample, the latter was dichotomized in higher (advanced technical professional, graduation from high school, college or university) vs. lower education (no educational qualifications, lower secondary school, secondary education). Further questions concerned the personal experience of bereavement (i.e. ‘Did you ever experience bereavement yourself?’ (yes/no), ‘How many losses did you experience within the last 2 years?’ (1, 2, 3,… 10, >10).

#### Prolonged grief symptoms

The ICG [37] was administered in its German version [38] among people who indicated that they had been bereaved (*N* = 763). The scale assesses indicators of disturbed grief with 19 items, such as anger, disbelief or non-acceptance on a 5-point Likert scale from never (0) to always (4). An example item is: ‘I think about this person so much that it is hard for me to do the things I normally do.’ The ICG’s internal consistency is excellent, as reported by Prigerson et al. [37]: α = .94 and by Lumbeck et al. [38]: α = .87. In our study, Cronbach’s alpha was .92

#### Public stigma: Attributions

Negative attributions were assessed by items previously used by Eisma [22] and Eisma et al. [23], which were selected based on research of public stigma in MDE [39], a German pilot study on stigma following bereavement, and research findings on personality characteristics that are commonly associated with grief severity [40, 41]. A back-translation method [42] was used to establish a German version. Respondents indicate on a 4-point Likert scale from completely disagree (1) to completely agree (4) to what extent they agree with the statement that the person described in the vignette is competent, warm, emotionally stable, dependent and sensitive.

#### Public stigma: Emotional reactions

Emotional reactions of respondents towards the person described in the vignette were assessed by the Emotional Reactions to Mental Illness Scale (ERMIS, [39]). It includes three stigma-related emotions, i.e. fear, anger and pity/compassion, measured with 3 items each. Pity is also referred to as prosocial emotion [43]. However, in previous studies addressing stigma in PGD and MDE, ‘fear’ and ‘pity’ yielded poor reliability [43]. Following Eisma et al. [23] we therefore used an adapted version. A back-translation method [42] was used to establish a German version. Fear was measured with 5 items (e.g. ‘He/She scares me.’), anger with 4 items (e.g. ‘I feel annoyed.’), and prosocial emotions were assessed with 4 items (e.g. ‘I feel pity.’). Respondents indicated their agreement with each statement on a 4-point Likert scale ranging from completely disagree (1) to completely agree (4). Average scores were obtained for each subscale. Eisma et al. [23] report good to acceptable internal consistencies for the scales (anger: α = .82; fear: α = .85; prosocial: α = .75). In the present study, internal consistencies were also good to acceptable for fear (α = .80) and prosocial emotions (α = .79), while anger demonstrated lower internal consistency (α = .64).

#### Public stigma: Social distance

The social distance scale according to Link et al. [44] was used to measure the respondents’ desire for social distance from the person described in the vignette (German version: [39]). The scale comprises seven items that represent different social relationships, e.g. ‘neighbor’. Using a 4-point Likert scale from completely disagree (1) to completely agree (4), respondents indicate to what extent they would, in the relationship presented, accept or not accept the person described in the vignette. A sum score is computed, ranging from 7 to 28. For the present analyses, the scale was inverted, so that higher scores indicate a stronger desire for social distance. Studies using the German version in depressive samples report good indices of reliability and validity [39, 45]. In the present study, internal consistency was good (α = .89).

### Manipulation check

After presentation of the vignettes, the first question of the manipulation check (‘The text describes a person who experiences severe difficulties in everyday life.’ [yes/ no]) ascertained whether respondents had correctly understood the level of impairment differentiating vignettes describing a person with clinically relevant PGD symptoms vs. integrated grief (no PGD symptoms). The second question ensured that respondents had correctly read the described diagnosis: ‘Please select the diagnosis the mental health professional gave to the described person.’ (prolonged grief disorder, depressive episode, schizophrenia, posttraumatic stress disorder, no diagnosis was given). After all other measures had been assessed, respondents indicated if they were familiar with the term PGD (‘Have you heard or read about PGD before?’ [yes/ no]).

### Data analysis

Prior to the main analyses, a randomization check for experimental group equivalence was performed on all background variables, using a combination of ANOVAs (for continuous variables) and χ^2^-tests (for categorical variables). To study the impact of the presence of a diagnosis and gender on stigmatizing reactions a 2 (Gender of bereaved person described: male vs. female) x 4 (Mental health condition: PGD symptoms and PGD diagnosis *vs*. PGD symptoms and MDE diagnosis *vs*. PGD symptoms and no diagnosis *vs*. no symptoms and no diagnosis) MANOVA was carried out with stigma indicators as dependent variables. Significant multivariate effects were followed by separate ANOVAs and effects were decomposed by planned contrasts (C):

C1: PGD symptoms and PGD diagnosis *vs* no symptoms and no diagnosis;
C2: PGD symptoms and MDE diagnosis *vs*. no symptoms and no diagnosis;
C3: PGD symptoms and PGD diagnosis *vs*. PGD symptoms and no diagnosis;
C4: PGD symptoms and MDE diagnosis *vs*. PGD symptoms and no diagnosis,
C5: PGD symptoms and PGD diagnosis *vs*. PGD symptoms and MDE diagnosis:

Assumption checks for the MANOVA detected 10 multivariate outliers. Since outliers may introduce bias into statistical estimates, they were excluded. For the main analyses, a two-sided significance level of 0.05 was used. For C1 and C2 (PGD symptoms and PGD/MDE diagnosis vs. no symptoms and no diagnosis) one-sided significance levels were used because those contrasts represented replication of the effect reported by Eisma [22]. To control for multiple testing in contrast analyses we used Bonferroni correction. Partial ƞ^2^’s were calculated, for which values of 0.01, 0.06 and 0.14 are viewed as small, medium and large effect sizes, respectively [46].

## Results

### Respondent characteristics

The sample size was determined via a-priori power analysis with GPower 3.1. Since we wished to be able to detect small differences between groups, we expected a small effect size of the hypothesized contrasts (expected ɳp^2^ = 0.01; power = .80). Thus, the power analysis indicated a required sample size of at least 788 respondents. In total, 997 respondents provided at least their demographics. Of those, 11% terminated participation before reaching the end of the survey (those cases were unsystematically distributed throughout the vignettes), leaving N = 885. One respondent was excluded because of implausible processing time. Twenty-two respondents answered both questions of the manipulation check incorrectly, indicating inadequate understanding of the vignettes. Values of 10 respondents were considered as multivariate outliers (Mahalanobis distance < .001; unsystematically distributed throughout vignettes). We excluded those cases, yielding a final sample size of *N* = 852. Missing data analysis demonstrated that missingness was < 1% for all items and scales.

Table 2 presents the distribution of respondents’ demographics and background variables across the different vignettes, i.e. experimental groups. In comparison to the general German population, respondents were younger (*M* = 32.6 vs. *M* = 45.80), and more often female (77% vs. 51%) [47], and individuals with a higher educational level (graduation from high school, college or university) were overrepresented (78% vs. 55%) [48]. Prolonged grief severity (ICG score) in participants who experienced bereavement was 16.10 (*SD* = 11.54), 153 respondents (18%) scored higher than 25. Respondents who score above the cut-off of 25 are considered at risk for PGD [37]. 29% of respondents indicated their familiarity with PGD.

**Table 2:** Demographics per experimental group (mental health condition X gender of bereaved person described in the vignette).

	PGD symptoms and PGD diagnosis		PGD symptoms and MDE diagnosis		PGD symptoms and no diagnosis		no symptoms and no diagnosis		
	Gender of bereaved person described								
*N*	male (108)	female (111)	male (105)	female (110)	male (99)	female (102)	male (109)	female (108)	Total (852)
Female (*N* (%))	84 (77.8)	88 (79.3)	81 (77.1)	83 (75.5)	76 (76.8)	83 (81.4)	77 (70.6)	87 (80.6)	659 (77.3)
Age in years	34.2	31.6	34.8	33.1	31.4	31.3	32.4	32.2	32.6
(*M* (*SD*))	(13.9)	(12.7)	(14.6)	(14.6)	(12.0)	(12.6)	(12.4)	(13.5)	(13.3)
Higher education (*N* (%))	80 (74.1)	85 (76.7)	78 (74.3)	87 (79.1)	80 (80.8)	83 (81.4)	91 (83.5)	81 (75.0)	665 (78.1)
Experience of bereavement within past 2 years (*N* (%))	49 (45.4)	53 (47.7)	54 (51.4)	60 (54.5)	45 (45.5)	41 (40.2)	47 (43.1)	54 (50.0)	403 (47.3)
Number of bereavements in lifetime (Median)	3	3	3	3	3	2	3	3	3
Relationship to deceased									
(*N* (%))									
Spouse	5 (4.6)	5 (4.5)	7 (6.7)	6 (5.5)	2 (2.0)	2 (2.0)	3 (2.8)	5 (4.6)	20 (2.3)
Child	3 (2.8)	5 (4.5)	2 (1.9)	1 (0.9)	1 (1.0)	2 (2.0)	5 (4.6)	1 (0.9)	35 (4.1)
Parent	24 (22.2)	21 (18.9)	27 (25.7)	19 (17.3)	17 (17.1)	21 (20.6)	28 (25.7)	27 (25.0)	184 (21.6)
Grandparent	41 (38.0)	40 (36.0)	40 (38.1)	41 (37.3)	48 (48.5)	37 (35.2)	35 (32.1)	40 (37.0)	322 (37.8)
Sibling	2 (1.9)	4 (3.6)	3 (2.9)	2 (1.8)	4 (4.0)	1 (1.0)	6 (5.5)	0 (0.0)	22 (2.6)
Other	21 (19.4)	24 (21.6)	15 (14.3)	29 (26.4)	18 (18.2)	28 (27.5)	21 (19.2)	24 (22.3)	180 (21.1)
ICG	16.3	16.8	16.1	17.7	14.0	16.0	16.0	15.7	16.1
(*M* (*SD*))	(12.4)	(12.2)	(12.1)	(11.4)	(9.3)	(9.8)	(12.5)	(12.0)	(11.5)

PGD = Prolonged Grief Disorder. MDE = Major Depressive Episode. *N* = sample size; *M* = mean; *SD* = standard deviation. ICG = Inventory of Complicated Grief. Higher education = advanced technical professional, graduation from high school, college or university. Missing data: For experience of bereavement within past 2 years there was one missing in the PGD symptoms and MDE diagnosis, female group. There were no significant differences detected on the demographic variables (all *p*s > .20).

### Randomization check

There were no significant differences between respondents in the eight different vignette groups on age *F*(7, 844) = 1.00, *p* = .43, gender, χ^2^(14) = 12.50, *p* = .57, education (higher vs. lower), χ^2^(14) = 12.44, *p* = .57, experience of bereavement χ2 (7) = .32, *p* = 1.0, bereavement experience within the past two years χ^2^(7) = 7.86, *p* = .35, and number of losses in lifetime χ^2^(70) = 76.38, *p* = .28. The number of people with clinically relevant levels of prolonged grief (ICG > 25) did also not differ between vignettes, (χ2 (7) = 10.68, *p* = .15).

### Main analysis

The MANOVA (mental health condition x gender of bereaved person described) yielded a significant large main effect for mental health condition, Roy's Largest Root = 2.76, *F*(9, 829) = 253.94, *p* < .01, ɳp^2^ = .73. This indicated a significant omnibus effect of mental health condition on respondents’ evaluations of the attribute items, emotional reactions and desire for social distance. Additionally, the MANOVA showed a small main effect of gender of bereaved person described, Roy's Largest Root = 0.03, *F*(9, 827) = 2.42, *p* = .01, ɳp^2^ = .03. Gender thus significantly influenced respondents’ overall evaluation on the dependent variables. The interaction of the factors did not significantly explain variance, Roy's Largest Root = 0.02, *F*(9, 829) = 1.54, *p* = .13, ɳp^2^ = .02. This non-significant interaction indicated that the overall effect of the factor mental health condition did not significantly differ depending on the gender of the bereaved person described, nor did the effect of gender of the bereaved person described differ depending on the different groups of mental health condition. Because of the statistical insignificance, the interaction effect was not followed up. Significant omnibus main effects were investigated in separate ANOVAs. Table 3 shows means and standard deviations of dependent variables for each vignette.

**Table 3:** Means and standard deviations of stigma outcomes per experimental group.

	PGD symptoms and PGD diagnosis		PGD symptoms and MDE diagnosis		PGD symptoms and no diagnosis		no symptoms and no diagnosis	
Gender of bereaved person described	Male	Female	Male	Female	Male	Female	Male	Female
in the vignette (*N)*	108	111	105	110	99	102	109	108
Attributes								
Competent	2.44 (0.70)	2.44 (0.73)	2.55 (0.75)	2.54 (0.80)	2.55 (0.77)	2.44 (0.61)	3.48 (0.50)	3.50 (0.56)
Warm	3.11 (0.69)	3.11 (0.67)	3.17 (0.70)	3.18 (0.70)	3.35 (0.63)	3.22 (0.59)	3.53 (0.54)	3.47 (0.55)
Dependent	2.86 (0.80)	2.95 (0.70)	2.79 (0.80)	2.78 (0.84)	2.90 (0.75)	3.07 (0.71)	1.61 (0.61)	1.47 (0.57)
Sensitive	3.48 (0.63)	3.43 (0.64)	3.48 (0.62)	3.44 (0.60)	3.43 (0.59)	3.47 (0.52)	3.03 (0.62)	2.99 (0.65)
Emotionally stable	1.44 (0.52)	1.57 (0.60)	1.54 (0.61)	1.52 (0.63)	1.59 (0.57)	1.59 (0.57)	3.50 (0.59)	3.45 (0.52)
Emotions								
Fear	1.86 (0.54)	1.80 (0.55)	1.69 (0.59)	1.92 (0.61)	1.86 (0.62)	1.82 (0.62)	1.40 (0.44)	1.40 (0.47)
Anger	1.32 (0.41)	1.37 (0.42)	1.30 (0.39)	1.34 (0.43)	1.32 (0.48)	1.39 (0.49)	1.20 (0.41)	1.22 (0.36)
Prosocial emotions	3.20 (0.57)	3.10 (0.53)	3.20 (0.54)	3.19 (0.50)	3.14 (0.58)	3.15 (0.58)	2.40 (0.64)	2.48 (0.67)
Social Distance^a^	15.26 (3.74)	14.21 (3.97)	14.63 (3.63)	14.10 (3.60)	14.56 (3.94)	14.20 (4.07)	10.90 (4.38)	9.72 (3.08)

PGD = Prolonged Grief Disorder. MDE = Major Depressive Episode. *N* = sample size.

^a^ = higher values indicate higher preferred social distance.

**Fig 1:**
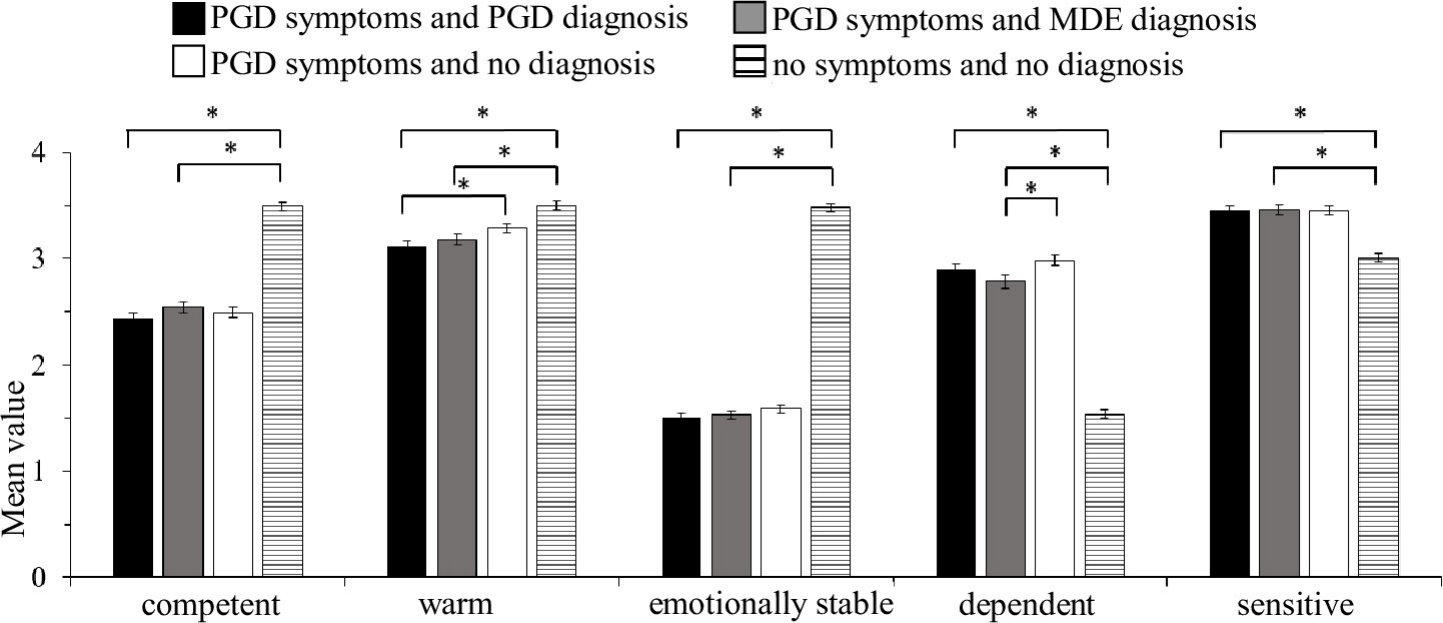
Mean scores and standard error of the mean value by mental health condition for attribute items. Note. Of the five a priori specified contrasts (C1-C5), only significant contrasts are indicated by brackets. (* adj. p < .01 (Bonferroni-Holm), *F*- Test).

**Fig 2:**
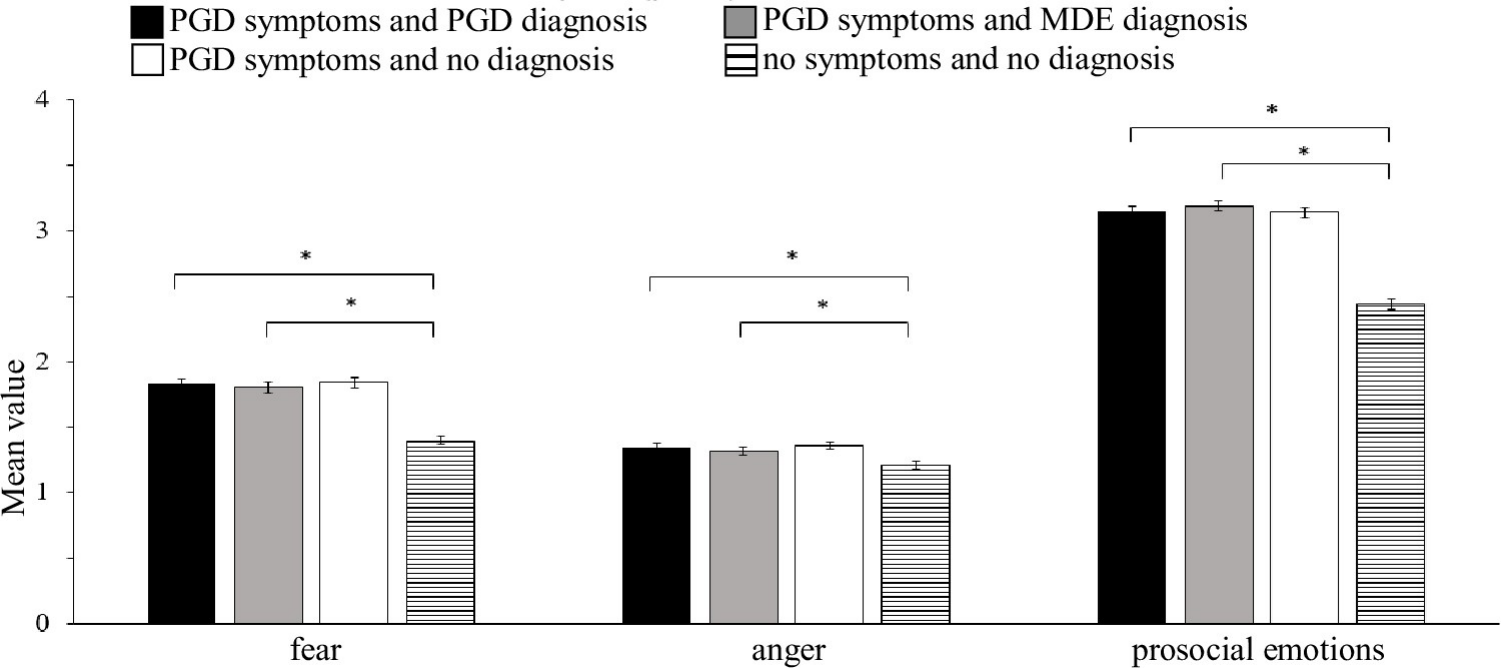
Mean scores and standard error of the mean value by mental health condition for emotional responses. Note. Of the five a priori specified contrasts (C1-C5), only significant contrasts are indicated by brackets. (* adj. *p* < .01 (Bonferroni-Holm), *F*- Test).

**Fig 3:**
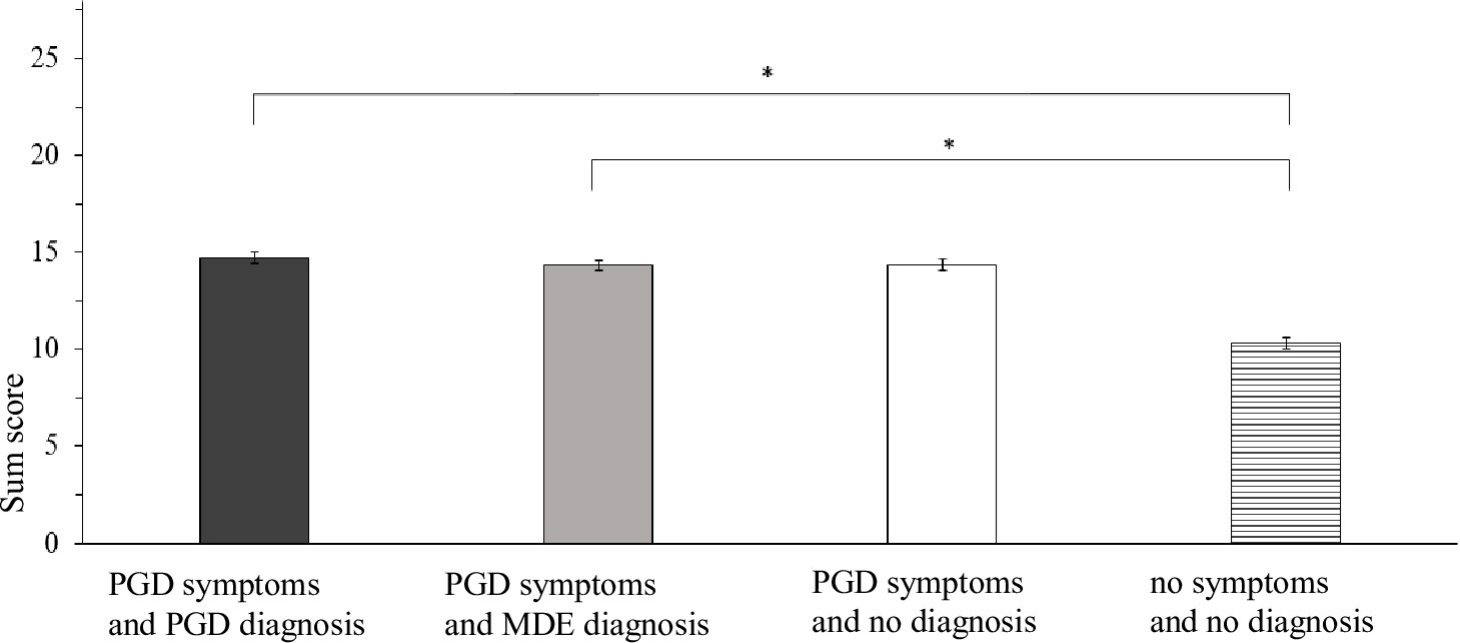
Mean scores and standard error of the sum score by mental health condition for preferred social distance. Note. Higher values indicate higher preferred social distance. Of the five a priori specified contrasts (C1-C5), only significant contrasts are indicated by brackets. (* adj. *p* < .01 (Bonferroni-Holm), *F*- Test).

Separate ANOVAs, each including both factors (mental health condition x gender of bereaved person described) were carried out to investigate significant omnibus MANOVA effects. For gender of the bereaved person described, no significant main effects were found on the attribute items and emotional reactions (all *F*s < 11.23, all *p*s > .11). However, a significant main effect of gender emerged for preferred social distance, *F*(1, 835) = 8.82, *p* = .003, ɳp^2^ = .01. Vignettes presenting a male bereaved person elicited higher preferred social distance than vignettes describing a female bereaved person (*M*_male_ = 13.80, *SD* = 4.30; *M*_female_ = 13.10, *SD* = 4.20). Consequently, the MANOVA omnibus effect for this factor was completely explained by the significant difference on preferred social distance between vignettes describing a male vs. female bereaved person. See S1 Table for exact statistics. The ANOVAs revealed main effects for mental health condition on all outcomes (all *F*s > 5.21, all *p*s < .001). To disentangle significant omnibus ANOVA effects of mental health condition planned contrasts were carried out. Table 4 shows the exact statistical results for the contrast analyses. Figs 1–3 show the mean scores and standard error of the mean value by mental health condition for the dependent variables. Significant contrasts are indicated by brackets and asterisks.

**Table 4:** Statistical results of the five planned contrasts for the stigma outcomes, i.e. attributes, emotional reactions and social distance.

	Significance *F*, *p*-value | ηp^2^				
	PGD symptoms and PGD diagnosis vs. no symptoms and no diagnosis^j^	PGD symptoms and MDE diagnosis vs. no symptoms and no diagnosis^j^	PGD symptoms and PGD diagnosis vs. PGD symptoms and no diagnosis	PGD symptoms and MDE diagnosis vs. PGD symptoms and no diagnosis	PGD symptoms and PGD diagnosis vs. PGD symptoms and MDE diagnosis
Attributes					
Competent^a^	252.75, < .001* |.23	201.39, < .001* |.19	0.66, = .42 |.001	0.579 = .45 |.001	2.56, = .11 |.003
Warm ^b^	40.91, *<* .001* |.05	27.40, *<* .001* |.03	8.00, = .01* |.01	2.940, = .87 |.003	1.26, = .26 |.001
Dependent ^c^	386.97, < .001* |.32	317.71, < .001* |.27	1.27, = .26 |.002	7.80,< .01* |.009	2.93, = .09 |.003
Sensitive ^d^	58.28, < .001* |.07	58.40, < .001* |.07	0.02, = .90 |.000	0.03, = .86 |.000	0.00, = .95 |.000
Emotionally stable^e^	1312.20, < .001* |.61	1255.74, < .001* |.60	2.53, = .11 |.003	1.12, = .29 |.001	0.29, = .59 |.000
Emotional reactions					
Fear^f^	64.32, < .001* |.07	55.65, < .001* |06	0.04, = .84 |.000	0.49, = .48 |.001	0.26, = .61 |.000
Anger ^g^	10.91, < .001* |.01	7.16, < .001* |.01	0.05, = .83 |.000	0.67, = .41 |.001	0.38, = .54 |.000
Prosocial emotions^h^	163.98, < .001* |.16	182.69, < .001* |.18	0.01, = .93 |.000	0.76, = .38 |.001	0.64, = .42 |.001
Social distance^i^	146.00, < .001* |.15	121.07, < .001* |.13	0.92, = .34 |.001	0.00, = .97 |.001	1.02, = .31 |.001

PGD = Prolonged Grief Disorder. MDE = Major Depressive Episode. ηp^2^ = effect size.

^a^ df = 1, 839.

^b^ df = 1, 840.

^c^ df = 1, 841.

^d^ df = 1, 840.

^e^ df = 1, 841.

^f^ df = 1, 843.

^g^ df = 1, 842.

^h^ 1, 842.

^i^ df = 1, 841.

^j^ = one-tailed.

*adj. *p* < .01 (Bonferroni-Holm)

Contrast 1 (PGD symptoms and PGD diagnosis *vs*. no symptoms and no diagnosis) and Contrast 2 (PGD symptoms and MDE diagnosis *vs*. no symptoms and no diagnosis) aimed to answer hypothesis 1: A person with PGD symptoms and a mental health diagnosis elicits more public stigma than a person with integrated grief. Group differences were significant for all dependent variables. These results indicate higher stigmatizing responses for vignettes describing either diagnosis (PGD and MDE) in combination with PGD symptoms when compared to integrated grief.

Contrast 3 (PGD symptoms and PGD diagnosis *vs*. PGD symptoms and no diagnosis) and contrast 4 (PGD symptoms and MDE diagnosis *vs*. PGD symptoms and no diagnosis) aimed to answer hypothesis 2: The public stigma towards a person with PGD symptoms depends on the presence of a mental health diagnosis (*vs*. no diagnosis). For contrast 3, results demonstrated no significant differences in the outcome variables with the exception of the attribute *warm*. Respondents rated persons with PGD symptoms and a PGD diagnosis as less warm than they did persons with PGD symptoms and no diagnosis. For contrast 4, there were also no significant differences in all outcome variables with the exception of the attribute *dependent*. Respondents rated persons with PGD symptoms and a MDE diagnosis as less dependent than they did persons with PGD symptoms and no diagnosis.

Contrast 5 aimed to answer hypothesis 3: When PGD symptoms are present public stigma differs between a PGD diagnosis and MDE diagnosis. Results demonstrated no significant differences in any outcome variables.

## Discussion

The WHO has recently introduced PGD as a new diagnosis. Researchers, practitioners and laypersons have repeatedly raised concerns about stigma. Indeed, previous research found that respondents reported more public stigma towards people with PGD *vs*. integrated grief [22, 23]. Yet, it was unclear if these effects could be attributed to the PGD label or PGD symptoms. This difference matters: a clinical diagnosis might facilitate treatment for those suffering from intense and prolonged grief. On the other hand—if the label elicits stigma- it might additionally burden these people. Using a vignette experiment, we cross-validated the studies of Eisma [22] and Eisma et al. [23]. Our main interest was to clarify if public stigma differs in response to a person with PGD symptoms and a mental health diagnosis *vs*. a person with PGD symptoms and no diagnosis. Additionally, we compared public stigma of a PGD diagnosis *vs*. MDE diagnosis (and symptoms) and investigated the effect of gender on public stigma and the aforementioned effects. This study is the first to investigate experimentally and separately the respective influences of PGD symptoms and PGD diagnostic label on stigmatization.

To sum up, while PGD symptoms paired with a PGD (or MDE) diagnosis were consistently associated with more public stigma compared to integrated grief, we found no robust additional effect of diagnostic labeling on public stigma when PGD symptoms were present. We also found no difference in public stigma between PGD symptoms and PGD diagnosis *vs*. PGD symptoms and MDE diagnosis. Gender of the bereaved person affected only preferred social distance. This effect was small.

More precisely, results showed hardly any differences in stigma between vignettes describing a person with PGD symptoms and a PGD diagnosis *vs*. a person with PGD symptoms and no diagnosis. Respondents’ answers differed neither in emotional reactions towards the person described in the vignette (*fear*, *anger and prosocial emotions*) nor in preferred social distance. Their answers also did not differ in attribute items (*competent*, *emotionally stable*, *dependent and sensitive*), except for *warm* and *dependent*: When described as suffering from PGD symptoms, a bereaved person with no PGD diagnosis was rated warmer than a person with PGD diagnosis and more dependent than a person with MDE diagnosis. Though statistically significant, both effects were small (ɳp^2^ = .01), amounting to a mean difference of only < 0.2 units on a 4-point Likert scale. Thus, in the presence of PGD symptoms the additional information that the bereaved person had been diagnosed with PGD or MDE by a mental health professional did not substantially affect public stigma. Our findings are in line with research on PGD’s nearest neighbors, MDE and PTSD, also showing no labeling effects [49, 50]. Complementing this interpretation and pointing to the importance of the presence of grief symptoms, the ecologically valid study of Johnson et al. [20] showed that people experiencing more intense grief perceive more negative reactions from friends and family.

Our finding is particularly important since previous research in PGD [22, 23] has not attempted to dismantle the effects of symptom presentation and diagnostic label on stigmatization. The present study’s results therefore deepen our understanding of stigmatization of PGD. They are also especially relevant in the light of various concerns that are associated with the introduction of PGD as new diagnostic category. In a recent survey Dietl, Wagner and Fydrich [51] reported that 25% of the respondents (German-speaking professionals in the fields of psychotherapy, psychology, counselling, medicine and palliative care) indicated that they considered it ‘quite likely or for sure’ that the introduction of PGD to ICD-11 will lead to an increased personal or social stigmatization of affected persons. Our vignette experiment, however, suggests that in the presence of PGD symptoms, symptoms themselves drive public stigma rather than the diagnostic label. However, when interpreting these findings, the public knowledge of PGD should be considered (for a detailed discussion: see the limitations section).

Further, we cross-validated Eisma`s [22] and Eisma et al.’s [23] findings: We found significant differences in public stigma variables between vignettes with PGD symptoms and a diagnosed mental health condition (PGD or MDE) *vs*. integrated grief (no PGD symptoms and no diagnosis). A person described in a vignette with PGD symptoms and a mental health condition was judged to be less competent, warm, emotionally stable and more dependent and sensitive. Respondents also indicated more fear, anger and prosocial emotions and a stronger desire for social distance towards a person with PGD symptoms and a mental health condition. Violated expectations of ‘correct grief responses’ may have contributed to this consistent effect. Previous research shows that intentions for social support (as opposite to stigmatization) depend on respondents’ perceptions of the appropriateness of the grief reaction [34]. People expect fewer grief-related symptoms and more recovery-related behavior over time [35]; the experience of severe grief reactions more than two years after loss is a clear violation of these assumptions leading to more negative social reactions.

Our third hypothesis aimed at exploring whether there is a difference in public stigma between PGD *vs*. MDE diagnoses when PGD symptoms are present. No differences emerged between the respective vignettes. Our findings can be situated in the literature comparing public stigma of PGD’s nearest neighbors, i.e. MDE and PTSD. Feldman and Candall [28] and Reavley and Jorm [25] found that PTSD elicited less stigma, possibly because the public attributes the cause (or ‘blame’) of PTSD to the seriousness and extraordinary nature of the external event and not to the person suffering from it [25]. Although PGD is also elicited by an external event, the stigma of PGD may still be more similar to that of MDE than that of PTSD, because bereavement is a universal experience: The public therefore might expect the cause for PGD to lie rather within the person. Supporting this hypothesized mechanism, Feldman and Candall [28] have shown that perceived personal responsibility for the mental health problem significantly predicts preferred social distance. Future research is needed to elucidate these possible mechanisms. Our results, however, suggest a preponderance of symptoms over labeling effects. In the presence of PGD symptoms, a PGD or MDE diagnosis does not elicit additional stigma, nor are there differences in public stigma for bereaved people with PGD symptoms and a PGD or MDE diagnosis.

Our last hypotheses 4) and 5) concerned the influence of the gender of the bereaved person, i.e. whether public stigma differs between vignettes that describe a male vs. female bereaved person. In our study, we found no significant differences for gender for any attributes or emotional reactions. This negative finding is in line with similar studies from the field of non-pathological grief [33, 35]. Our study had sufficient statistical power to detect respective effects and our results thus corroborate the finding that these indicators of stigma do not vary with the gender of the bereaved person and extend the investigation of gender effects on these stigma indicators to pathological grief.

For the behavioral component of public stigma, on the other hand, we found that preferred social distance was relatively higher towards bereaved men than women. When interpreting this finding, however, its small absolute magnitude (ɳp^2^ = .01) and the characteristics of our sample need to be taken into account. In our sample, female participants formed the majority. It is possible that female participants felt more sympathy for female grievers and indicated a lower preferred social distance towards them because of social proximity. Additionally, previous research has also demonstrated that gender effects on preferred social distance towards a bereaved person may be qualified by both: the grief severity of the bereaved person [32] and the cause of death [31]. Concerning grief severity, Kubitz et al. [32] found that in the case of non-pathological grief, participants were less willing to interact with men than women only if grief severity was high. In contrast, in our study the effect of gender of the bereaved on preferred social distance was independent of the presence or absence of PGD symptoms and label. Differences both in the respective operationalization of social distance and in the age of the bereaved person described in the vignette (Kubitz et al. [32]: early adulthood) may contribute to these contrasting findings. Concerning the role of cause of death, Penman et al. [31] reported that a vignette describing a male bereaved person elicited a stronger desire for social distance only when the death of the partner was caused by a sudden, natural cause; i.e. stroke. It could be that our findings align with this study, because stroke was uniformly the type of death in our vignettes.

### Limitations and future directions

This is the first study on public stigma in PGD in a German sample. Its major strengths lie in qualities that contribute to its internal validity and methodological rigor. Its design built on previous findings [22, 23] in order to manipulate the relevant constructs in a well-controlled experiment with a manipulation check. Thus, our study was able to disentangle previously reported effects and elucidate the role of diagnostic labeling. Its pre-calculated large sample size allowed us to detect small effects with adequate power and have confidence in non-significant results. Additionally, while previous research on stigma in bereavement has often used ad-hoc items (e.g. [33]) or focused only on selected indicators of stigma (e.g. only social distance [31]), our study measured different components of public stigma with well-established scales. By using the most recently established PGD-criteria in our vignettes, our results complement previous research [31] with evidence from ICD-11 criteria.

However, some study limitations merit comment: First, female participants with higher education were overrepresented in the sample, which poses a threat to the external validity of our findings. The sample composition may be due to the overrepresentation of female participants in bereavement research in general [52], the use of convenience sampling, and some of our various recruitment strategies (e.g., contacting university mailing lists). However, the high degree of consistency between our main findings on PGD and stigma with the results from prior studies in community samples from different countries [21–23], also suggests that our findings generalize to different populations. To explore if the various recruitment strategies attract participants with different characteristics, future research could assess by which channel each respondent was recruited. To minimize sampling bias in this field of research, future studies should aim to recruit a more diverse or ideally population-representative sample, e.g. by specifically recruiting male participants. Second, the reliability of the anger scale was relatively low. This might partly be due to the brevity of the scale and the lack of robustness of Cronbach's alpha to the number of items in a scale [53]. Future studies should aim to improve the internal consistency of this subscale possibly by increasing the length of the scale. Third, while the vignette method gave our experiment high internal validity, it also restricted its external validity. The vignettes included very little information on the bereaved person besides the PGD symptoms. If respondents had actually known the bereaved person, they may have felt more empathy and consequently reacted differently. Nevertheless, our results match with the externally valid study of Johnson et al. [20] who reported that there was a significant association between participants’ grief symptom severity and the number of their actual or expected negative reactions from friends and family. Generally, the vignette method is a well-accepted and prominent method to study public stigma [54]. Still, future studies should apply different methodological approaches to shed light on public stigma in PGD. Lastly, since PGD is a new diagnostic category, the public may not yet have a clear concept or stereotypes about PGD as is the case in other mental illnesses. This lack of familiarity could have limited the stigmatizing effect of the PGD label. In fact, only 29% of the respondents indicated that they were familiar with the diagnosis PGD. In an attempt to control for the influence of familiarity with the label, we compared public stigma between the new PGD label and the well-known MDE label and found no differences in stigma between the conditions, when PGD symptoms were present. However, we did not test if the label in and of itself could elicit public stigma as too few participants may be familiar with it. Once such knowledge becomes more common, the label PGD itself could come to represent the symptoms that it encompasses and thereby elicit stigma, especially if the label is the only available information about someone.

Our findings contribute to the present understanding of stigma in PGD and suggest potential directions for future research. First, an interesting avenue for future research could be to test if the label PGD has a stigmatizing effect in and of itself. Building on research in the field of stigma towards PTSD [27], future studies could investigate this ‘pure’ labeling effect of a PGD diagnosis. It seems especially relevant to conduct this research with both PGD ‘naive’ respondents who have little knowledge of the diagnosis and respondents who may have a clearer concept (and potentially more stigma-relevant knowledge) of PGD. Second, while the experimental design of our study controlled for potential effects of respondents’ sociodemographic characteristics on public stigma, it would be interesting to investigate which characteristics of the respondents contribute to public stigma towards bereaved persons. Such variables could include one’s own bereavement experiences, previous traumatic experiences, occupational situation, socio-economic status, or personal status of the respondents. Third, researchers could cross-validate our findings using different methodological approaches such as complementing our assessments with the Implicit Association Test [55] or using video vignettes instead of written material. Fourth, long-term studies should address the question how public stigma towards persons with PGD translates into self-stigma of affected persons and eventually influences their symptoms and other mental health outcomes. Lastly, one way to target stigma reduction could be to treat PGD, with reduced symptomatology presumably eliciting less public stigma. Drawing on extended contact hypothesis, another approach can be to create a more responsive environment using informational campaigns [56, 57].

## Conclusions

Our study is the first to show that labeling PGD symptoms with a grief-specific diagnosis does not produce public stigma in addition to that caused by the symptoms. Our findings thus contribute to the ongoing debate by researchers and lay people who fear that the introduction of PGD as diagnostic category may cause stigma and therefore additionally burden affected people [13, 22, 51, 58]. While we acknowledge these concerns, our experimental results show no indication of such an additional effect despite adequate statistical power. Yet, as we have pointed out, stigmatization may evolve with familiarity towards a new diagnosis. While the vignette-based experiment is well-established in stigma research [54] because it affords a high degree of standardization, and our specific research questions could not have been addressed within a real-life context, experimental evidence is always limited in its external validity. The generalizability of our results therefore remains to be tested. It is also essential to enrich this line of research on stigma and PGD in the future by coupling it with evidence from observational studies in bereaved samples: Johnson’s et al. (2009) observational study provides support that stigma might be elicited by symptoms of PGD.

In sum, public stigma seems to be stronger towards individuals with prolonged grief symptoms than towards those with integrated grief. This highlights a potential benefit of accepting PGD as an official diagnosis: it is likely to increase the chance that affected persons receive specific and adequate treatment for prolonged grief, which is proven to reduce PGD symptoms [59]. In addition to alleviating individual suffering, reducing PGD severity might then contribute to a decrease in stigmatizing reactions from the social environment. Additionally, PGD treatments might strive to enhance social acceptance and support of the bereaved person to reduce experienced stigma and its potential negative consequences.

## Supporting information

S1 Table ANOVA results for each outcome variable. ANOVA, analysis of variance. ηp^2^ = effect size. MHC = mental health condition. ^a^*df* = 3,835. ^b^ *df* = 1,835. ^c^ *df* = 3,835. *p < .05, ** *p* < .01, ***, *p* < .001.
(PDF)
