# Supplementary material for: Public stigma towards prolonged grief disorder: Does diagnostic labeling matter?
Source: PLoS One. 2020 Sep 11;15(9):e0237021. doi: 10.1371/journal.pone.0237021 (PMC7485774; doi:10.1371/journal.pone.0237021)
Supplement: S1 Table — ANOVA, analysis of variance. ηp2 = effect size. MHC = mental health condition. adf = 3,835. b df = 1,835. c df = 3,835. *p < .05, ** p < .01, ***, p < .001. (PDF) [file pone.0237021.s001.pdf]

S1 Supporting information.

S1 Table. ANOVA results for each outcome variable.

	ANOVA F, |η^2		
	MHC[a]	Gender[b]	MHC x Gender[c]
Attributes			
Competent	113.23*** |.23	0.26 |.000	.41 |.001
Warm	15.55*** |.05	1.10 |.001	.56 |.002
Dependent	189.63*** |.40	0.33 |.000	1.70 |.006
Sensitive	28.63*** |.09	0.25 |.000	.25 |.001
Emotionally stable	624.47*** |.69	0.16 |.000	1.08 |.004
Emotional reactions			
Fear	31.11*** |.10	0.64 |.001	3.00* |.011
Anger	5.21** |.02	2.50 |.003	.08 |.000
Prosocial emotions	84.46*** |.23	0.03 |.000	.86 |.003
Social distance	65.12*** |.19	8.82** |.01	.56 |.002

Note. ANOVA, analysis of variance. ηp^2 = effect size. MHC = mental health condition.

[a] df = 3,835. [b] df = 1,835. [c] df = 3,835.

*p < .05, ** p <.01, ***, p <.001
